# Editorial: Genetics of familial hypercholesterolemia: New insight—Volume II

**DOI:** 10.3389/fgene.2022.1041342

**Published:** 2022-10-12

**Authors:** Alpo Vuorio, Uma Ramaswami, Kirsten B. Holven

**Affiliations:** ^1^ Mehiläinen Airport Health Centre, Vantaa, Finland; ^2^ Department of Forensic Medicine, University of Helsinki, Helsinki, Finland; ^3^ Lysosomal Disorders Unit, Royal Free London National Health Service Foundation Trust, London, United Kingdom; ^4^ Genetics and Genomics Department, University College London, London, United Kingdom; ^5^ Department of Nutrition, Institute of Basic Medical Sciences, University of Oslo, Oslo, Norway; ^6^ Norwegian National Advisory Unit on Familial Hypercholesterolemia, Department of Endocrinology, Morbid Obesity and Preventive Medicine, Oslo University Hospital, Oslo, Norway

**Keywords:** familial hypercholesterolemia, mutations, LDL, Lp(a), statins, PCSK9 inhibitors, COVID-19

The second volume of Research Topic “Genetics of Familial hypercholesterolemia: New Insight—Volume II” attracted nearly 100 authors from 16 countries to publish their research articles and again we achieved our goal of bringing together researches of familial hypercholesterolemia (FH) worldwide and to increase FH related awareness of genetics, diagnostics, and risk factors. In addition, Research Topic like future therapies for the treatment of homozygous form of FH (HoFH) gene therapy are discussed in this Research Topic (Li and Wu).

The systematic review and meta-analysis in this Research Topic by Toft-Nielsen et al. showed that heterozygous familial hypercholesterolemia (HeFH) is a common genetic disease with an estimated prevalence of about 1 in 300 persons meaning that over 25 million individuals are affected by HeFH worldwide. Interestingly in this meta-analysis estimated FH prevalence varied across ethnicity and ranged from 1:400 to 1:192 being highest among black and brown individuals. Unfortunately, HeFH has remained an underdiagnosed and undertreated disease (Representatives of the Global Familial Hypercholesterolemia Community, 2020).

While it is important to know the worldwide prevalence of FH it is also important to know the local prevalence of FH in different countries. In this Research Topic (Diboun et al.) for the first time found out the prevalence of HeFH in Qatar. Their results revealed an estimated prevalence of HeFH in Qatar to be 0.8% (1:125) for definite/probable cases. The two most common mutations in Qatar were in *LDLR* but additionally it was found mutations also in *APOB* and *PCSK9.* No HoFH patients were found in this study carried out in a Qatar. In a study by (Rimbert et al.) the prevalence of genetically confirmed HeFH was studied in an Emirati population sample. Rimbert and co-authors (2022) recruited 229 patients with serum low-density cholesterol (LDL-C) over the 95th percentile and used next generation sequencing to screen *LDLR, APOB, PCSK9* and *LDLRAP1.* In this study, the prevalence of genetically confirmed HeFH was 7% with marked hypercholesterolemia as determined by correcting LDL-C for the use of lipid-lowering treatment. Rimbert et al. argue that the results of this study are helpful when planning to cascade screening programs in the United Arab Emirates.

Finding new mutations causing FH remains important because in the majority of countries FH remains vastly underdiagnosed and only about 1% or less of the potential FH cases have been found (Nordestgaard et al., 2013). In a study by Hu et al. the whole-exome sequencing and Sanger sequencing revealed two *LDLR* missense mutations *LDLR* c.226 G > C and c.1003 G > T in one family, causing most probably LDLR uptake dysfunction. Hu and co-authors (2021) remind us that finding new FH-causing mutations helps to carry out diagnostic screening at population level and start early prevention of vascular disease. In fact, a recent recommendation underscores that especially screening of paediatric FH patients is efficient and cost-effective in preventing vascular disease (Gidding et al., 2022).

Tada et al. studied missense variants in HeFH patients (which can estimate residual LDLR activity) and protein truncating variants (PTVs), which may have lost their LDL function. Based on variant data the authors analysed the occurrence of major adverse cardiac events (MACE). Both PTVs and missense variants were significantly associated with MACEs (hazard ratio [HR] = 1.58, 95% confidence interval [CI] = 1.08–2.08, *p* = 0.0033 and HR = 3.24, 95% CI = 2.12–4.40, *p* = 3.9 × 10^–6^, respectively). The authors conclude that genetic testing is useful to further stratify the risk among HeFH patients.

Gratton et al. discuss in their article the importance of polygenic cause for elevated LDL-C. The diagnosis of polygenic hypercholesterolemia is in this study based on a 12-single nucleotide polymorphism (SNP) score and this score was evaluated among South Asian (SA) and Black and Caribbean (BC) ethnicities. Gratton and co-authors (2022) found differences in LDL-C and 12-SNP score distribution between ethnicities. The authors conclude that there is a need to carry more research to find out benefits and risks of returning polygenic information to patients.

The role of registers improving management of HeFH children was showed by (Gazzotti et al.). In this Italian LIPIGEN register study analysis of about 1,600 HeFH children were analysed, and untreated low-density lipoprotein cholesterol (LDL-C) was about 220 mg/dl which in other European HeFH children’s cohorts is on a range of LDL-C 188–220 mg/dl (Futema et al., 2020). Importantly Futema et al., 2020 showed in their European countries analysis that the age of FH diagnosis varies significantly across countries and alarmingly almost three-quarters of the over 10-year-old untreated HeFH children have their LDL-C over the treatment target limit. This result by Futema and co-authors (2020) is particularly unfortunate because statins has shown to be an effective and safe treatment for HeFH in children (Luirink et al., 2019; Vuorio et al., 2019).

In recall-by genotype (RbG) studies, people who carry genotypes of special interest are recalled from a biobank for more detailed investigations. In an Estonian Biobank (EstBB) RbG long-term follow up study on lipid lowering treatment (LLT) adherence of 34 recalled HeFH patients was compared to 291 controls having the same mutations causing HeFH (Nurm et al.). It was shown in this study that the recalled group had significantly more LLT users compared to the non-recalled group (79% vs. 53%, *p* < 0.005). This HeFH RbG study showed positive impact on LLT treatment among EstBB participants.

(Soufi et al.) demonstrated in their study the advantages of Oxford Nanopore technology sequencing compared to Sanger and NextGen sequencing regarding FH diagnostics. Based on their experience, Oxford Nanopore technology sequencing is easy to operate, low-cost, and allows parallel sample sequencing. As an example of costs, they mention the following comparison. Genetic analysis of the *LDLR* of one patient would cost $ 1.060 using Sanger sequencing and $890 using Next Gen sequencing while using the new Nanopore technology the cost is only $ 109.

Recent studies have shown that among FH patients elevated lipoprotein (a) [Lp(a)] is associated with an increased risk of atherosclerotic vascular disease (ASCVD) (Bogsrud et al., 2019; Vuorio et al., 2020; Kronenberg et al., 2022). In a very large Hungarian population-based study, Németh et al. included 590,500 patients and used the Dutch Lipid Clinic Network scores and machine learning to identify probable and definite HeFH patients. They found 459 HeFH patients of which 221 had serum Lp(a) measurements available. Patients with HeFH had significantly higher serum Lp(a) levels compared to non-FH subjects [236 (92.5; 698.5) vs 167 (80.2; 431.5) mg/L, *p* < 0.01]. Additionally, 35% of HeFH patients had serum Lp(a) levels over 500 mg/L. Atherosclerotic complications were more often present in FH patients compared to non-FH subjects (46.6 vs 13.9%). Currently lowering serum Lp(a) by about 30% is possible by using proprotein convertase subtilisin/kexin type 9 (PCSK9) inhibitors (Spolitu et al., 2019). New drugs, such as antisense oligonucleotides against Lp(a) and small interfering RNA lower serum Lp(a) by about 80–90% (Vuorio et al., 2020).

Because Lp(a) has shown to be a crucial risk factor for FH patients it is also important to diagnose those FH patients having elevated serum Lp(a). To this end, Loh et al. suggest an assessment of Lp(a) as a part of FH cascade screening. They have estimated that those probands with HeFH and hyper-Lp(a), the yield of detection of hyper-Lp(a) is approximately 1 individual for every 2.1–2.4 relatives tested. The yield to find both HeFH and high Lp(a) is 1 individual for every 3–3.4 relatives screened. Therefore, the combined screening for FH and Lp(a) is very promising (Chakraborty et al., 2022).

There are two retrospective studies in this Research Topic in which a group of 1,236 HeFH patients (841 women; 395 men; and 243 relatives; mean age 44.8 ± 16.7) were followed over 50 years in the General University Hospital in Praque (Altschmiedova et al.; Todorovova et al.). Todorova and co-authors (2022) found that during the follow-up 50 years decrease of serum LDL-C levels was more than 50% when compared to the initial LDL-C values in the beginning of the follow-up in this Czech Lipid Clinic study. Altschmiedova and co-authors (2022) found in this same HeFH group of patients that serum baseline Lp(a) and triglycerides had a strong correlation with ASCVD. Based on these two retrospective analyses authors conclude that HeFH patients benefit from follow-up and treatment in specialized lipid centers.

Follow-up studies of severe acute respiratory syndrome (SARS) caused by the coronavirus (SARS-CoV) epidemic between 2002 and 2003 show deterioration of general health during 24 months after the infection (Ngai et al., 2010; Zhang et al., 2020). Additionally, lipid and glucose metabolism may also remain altered for a long time after a SARS-CoV infection (Wu et al., 2017). In FH with COVID-19 the pre-existing endothelial dysfunction could increase long-term vascular complications (Vuorio et al., 2021). So far it has been already found that COVID-19 illness increases cardiovascular risk of HeFH patients with or without ASCVD (Myers et al., 2021). In their opinion article, Vuorio et al and co-authors (2022) express concern about FH patients who have suffered SARS-CoV-2 infection because post-infection hypercoagulable states may persist even longer among patients with FH compared to non-FH patients. This concern is based on the notion that FH endothelium in these patients is exposed to a lifelong high serum LDL-C and often also to elevated serum Lp(a) levels, which jointly worsen endothelial dysfunction (Vuorio et al., 2021). Therefore, the clinicians need to consider the effective LLT in FH patients during and after a SARS-CoV-2 infection.

## References

[B1] Bogsrud M. P. Græsdal A. Johansen D. Langslet G. Hovland A. Arnesen K. E. (2019). LDL-cholesterol goal achievement, cardiovascular disease, and attributed risk of lp(a) in A large cohort of predominantly genetically verified familial hypercholesterolemia. J. Clin. Lipidol. 13 (2), 279–286. 10.1016/j.jacl.2019.01.010 30910667

[B2] Chakraborty A. Pang J. Chan D. C. Ellis K. L. Hooper A. J. Bell D. A. (2022). Cascade testing for elevated lipoprotein(a) in relatives of probands with familial hypercholesterolaemia and elevated lipoprotein(a). Atherosclerosis 349, 219–226. 10.1016/j.atherosclerosis.2021.11.004 34862044

[B3] Futema M. Ramaswami U. Tichy L. Bogsrud M. P. Holven K. B. Roeters van Lennep J. (2020). Comparison of the characteristics at diagnosis and treatment of children with heterozygous familial hypercholesterolaemia (FH) from eight European countries. Atherosclerosis 292, 178–187. 10.1016/j.atherosclerosis.2019.11.012 31809987 PMC6949888

[B4] Gidding S. S. Wiegman A. Groselj U. Freiberger T. Peretti N. Dharmayat K. I. (2022). Paediatric familial hypercholesterolaemia screening in europe – public policy background and recommendations. Eur. J. Prev. Cardiol., zwac200. 10.1093/eurjpc/zwac200 36059237

[B5] Kronenberg F. Mora S. Stroes E. S. G. Ference B. A. Arsenault B. J. Berglund L. (2022). Lipoprotein(a) in atherosclerotic cardiovascular disease and aortic stenosis: A European atherosclerosis society consensus statement. Eur. Heart J. 29, ehac361. Epub ahead of print. PMID: 36036785. 10.1093/eurheartj/ehac361 PMC9639807 36036785

[B6] Luirink I. K. Wiegman A. Kusters D. M. Hof M. H. Groothoff J. W. de Groot E. (2019). 20-Year follow-up of statins in children with familial hypercholesterolemia. N. Engl. J. Med. 381 (16), 1547–1556. 10.1056/NEJMoa1816454 31618540

[B7] Myers K. D. Wilemon K. McGowan M. P. Howard W. Staszak D. Rader D. J. (2021). COVID-19 associated risks of myocardial infarction in persons with familial hypercholesterolemia with or without ASCVD. Am. J. Prev. Cardiol. 7, 100197. 10.1016/j.ajpc.2021.100197 34611637 PMC8387291

[B8] Ngai J. C. Ko F. W. Ng S. S. To K. W. Tong M. Hui D. S. (2010). The long-term impact of severe acute respiratory syndrome on pulmonary function, exercise capacity and health status. Respirology 15 (3), 543–550. 10.1111/j.1440-1843.2010.01720.x 20337995 PMC7192220

[B9] Nordestgaard B. G. Chapman M. J. Humphries S. E. Ginsberg H. N. Masana L. Descamps O. S. European Atherosclerosis Society Consensus Panel, etal (2013). Familial Hypercholesterolaemia Is underdiagnosed and undertreated in the general population: Guidance for clinicians to prevent coronary heart disease: Consensus statement of the European atherosclerosis society, Eur. Heart J., 34(45), 3478–90a. 10.1093/eurheartj/eht273 23956253 PMC3844152

[B10] Spolitu S. Dai W. Zadroga J. A. Ozcan L. (2019). Proprotein convertase subtilisin/kexin type 9 and lipid metabolism. Curr. Opin. Lipidol. 30 (3), 186–191. 10.1097/MOL.0000000000000601 30925519 PMC6824479

[B11] Vuorio A. Kuoppala J. Kovanen P. T. Humphries S. E. Tonstad S. Wiegman A. (20192019). Statins for children with familial hypercholesterolemia. Cochrane Database Syst. Rev. 11, CD006401. 10.1002/14651858.CD006401.pub5 PMC6836374 31696945

[B12] Vuorio A. Strandberg T. E. RaalSantos F. R. D. Kovanen P. T. (2021). Familial hypercholesterolemia and COVID-19: A menacing but treatable vasculopathic condition. Atheroscler. Plus 43, 3–6. 10.1016/j.athplu.2021.08.001 34622243 PMC8349422

[B13] Vuorio A. Watts G. F. Schneider W. J. Tsimikas S. Kovanen P. T. (2020). Familial hypercholesterolemia and elevated lipoprotein(a): Double heritable risk and new therapeutic opportunities. J. Intern. Med. 287 (1), 2–18. 10.1111/joim.12981 31858669

[B14] Representatives of the Global Familial Hypercholesterolemia Community Wilemon K. A. Patel J. Aguilar-Salinas C. Ahmed C. D. Alkhnifsawi M. Almahmeed W. (2020). Reducing the clinical and public health burden of familial hypercholesterolemia: A global call to action. JAMA Cardiol.JAMA Cardiol. 55 (25), 217–229. PMID: 31895433. 10.1001/jamacardio.2019.5173 31895433

[B15] Wu Q. Zhou L. Sun X. Yan Z. Hu C. Wu J. (2017). Altered lipid metabolism in recovered SARS patients twelve years after infection. Sci. Rep. 7 (1), 9110. 10.1038/s41598-017-09536-z 28831119 PMC5567209

[B16] Zhang P. Li J. Liu H. Han N. Ju J. Kou Y. (2020). Long-term bone and lung consequences associated with hospital-acquired severe acute respiratory syndrome: A 15-year follow-up from a prospective cohort study. Bone Res. 8, 8. 10.1038/s41413-020-0084-5 32128276 PMC7018717

